# Phenotypic impacts of treatment-selected mutations in HIV-2 protease on darunavir and lopinavir susceptibility: Evaluating genotypic HIV-2 drug resistance tools

**DOI:** 10.1371/journal.pgph.0005743

**Published:** 2026-02-04

**Authors:** Dana N. Raugi, Robert S. Nixon, Robert A. Smith, Stephen E. Hawes, Khardiata Diallo, Mouhamadou Baïla Diallo, Khadim Faye, Binetou Diaw, Marie Pierre Sy, Fatima Sall, Moussa Seydi, Geoffrey S. Gottlieb, for the University of Washington-Senegal HIV-2 Study Group

**Affiliations:** 1 University of Washington, Departments of Medicine Division of Allergy and Infectious Diseases, Center for Emerging and Re-Emerging Infectious Diseases, Seattle, Washington, United States of America; 2 Epidemiology, University of Washington, Seattle, Washington, United States of America; 3 Global Health, University of Washington, Seattle, Washington, United States of America; 4 Service des Maladies Infectieuses et Tropicales, Ibrahima Diop Mar, Centre Hospitalier National Universitaire de Fann, Universite Cheikh Anta Diop de Dakar, Dakar, Senegal; National University of Asuncion: Universidad Nacional de Asuncion, PARAGUAY

## Abstract

Compared to HIV-1, HIV-2 infection is characterized by lower viral loads and slower decline in CD4 cells, however the majority of people living with HIV-2 (PLWH2) progress to AIDS and will benefit from antiretroviral therapy. Mutations leading to protease inhibitor (PI) resistance in HIV-2 are poorly characterized, but have important implications for second-line therapy. We evaluated the phenotypic drug susceptibility impacts of HIV-2 protease changes which are identified in genotypic resistance tools. We generated a library of 54 full length HIV-2_ROD9_ clones that included 21 individual protease mutations, alone or in various combinations. We generated eight additional clones containing combinations of changes observed in PI-treated PLWH2. We tested the clones in a single-cycle PI assay to determine darunavir (DRV) and lopinavir (LPV) EC_50_, and calculated fold change resistance relative to wild-type HIV-2_ROD9_. Four of the 21 amino acid changes tested alone conferred PI resistance: V47A and T56V conferred 4.1 and 2.9-fold resistance, respectively, to LPV, I50V conferred 4.6-fold resistance to DRV, and I54M conferred 5.5-fold resistance to DRV and 2.2-fold resistance to LPV. Other changes either lowered the EC_50_ or caused no change. Some combinations including V47A, I50V, I54M, or T56V also conferred resistance, with EC_50_ values 4.4 to 17-fold higher than wild-type. Six of eight PLWH2-derived strains were replication-competent: five exhibited resistance to LPV (>8.8-fold resistance), and three exhibited resistance to DRV (>4.7-fold). HIV-1 and HIV-2 are not equivalently susceptible to all antiretroviral agents and do not utilize identical pathways to resistance. We provide phenotypic evidence supporting the resistance role of changes in HIV-2 protease which do not have HIV-1 analogues, as well as evidence that analogues of “major” resistance changes in HIV-1 may have no resistance impacts in HIV-2, despite apparent treatment selection. These results should inform the HIV-2 genotypic resistance tools and help improve treatment for PLWH2.

## Introduction

The UNAIDS’ 95-95-95 targets are directed predominantly towards control of human immunodeficiency virus type 1 (HIV-1), however, any effort to end the HIV/AIDS pandemic must also include HIV-2, which affects one to two million people primarily in West Africa and countries with socio-economic ties to the region [1]. Although HIV-2 causes a clinical syndrome similar to that seen in infection with HIV-1, the majority of people living with HIV-2 (PLWH2) experience a slower disease course, characterized by longer asymptomatic period, lower and frequently undetectable plasma viral loads, and slower decline in CD4 cell counts [2,3]. Despite this difference, in the absence of effective antiretroviral therapy (ART), a significant proportion of those infected will go on to develop clinical disease and are at risk for AIDS-related mortality [4].

Despite nearly 40 years since the discovery of HIV-2 [5], treatment for HIV-2 infection lags far behind treatment for HIV-1 [6], with only four clinical trials of ART for HIV-2 (three single-arm, one randomized) having been conducted [7–10]. As a result, national and international guidelines for HIV-2 treatment are based largely on observational cohort data, *in vitro* studies, and extrapolation from HIV-1. Data to guide second-line therapy are scarce, and genotypic drug resistance testing is generally limited to academic research settings (including Research Use Only assays), with resources to interpret genotypic testing that are inadequately supported by robust clinical datasets and phenotypic testing. From ~1998 until ~2019, protease inhibitor (PI) -based therapy was the standard of care for PLWH2 in resource-limited settings (RLS) [11], based largely on the intrinsic resistance of HIV-2 to non-nucleoside reverse transcriptase inhibitors (NNRTI) [12], the widespread availability of PI for programmatic second-line therapy for PLWH1, and the lack of access to integrase strand transfer inhibitors (INSTI).

In 2018, the World Health Organization issued new guidelines for the treatment of HIV [13], which recommended dolutegravir (DTG), tenofovir disoproxil fumarate, and lamivudine (or emtricitabine) as first-line ART for all persons living with HIV (PLWH), regardless of HIV type. The same combination was recommended for second-line therapy for PLWH failing non-DTG-based regimens. Thus, a campaign began to roll out DTG-based ART, specifically tenofovir-lamivudine-DTG (TLD), for programmatic ART worldwide, partly because it appears effective against all types of HIV infection (HIV-1, HIV-2, and HIV-1/2 dual infection), and additionally because clinical trials for TLD in people living with HIV-1 (PLWH1) suggested a high barrier to DTG resistance [14–18]. However, recent data have suggested that emergence of DTG resistance is more common than once-hoped [19], particularly among patients switching to TLD with unsuppressed viral loads [20]. The DTG RESIST study in sub-Saharan Africa recently reported that among PLWH1 with viremia (plasma viral load >1000 copies/mL) receiving DTG-based ART, 26.0% had at least one major drug resistance mutation in integrase [21]. To date, no studies have examined drug resistance in TLD-treated PLWH2 in sub-Saharan Africa, however, for PLWH2, TLD failure in RLS would necessitate a switch or return to PI-based ART [22]. For this reason, a better understanding of PI resistance in HIV-2 remains vital.

Three genotypic resistance tools exist for HIV-2. Two, Rega and HIV2EU, implemented in HIV-GRADE (available at https://www.hiv-grade.de/HIV2EU/deployed/grade.pl?program=hivalg), are based on case reports, cohort analyses, scant available phenotypic data (including serial passage experiments), and expert opinion (available at https://rega.kuleuven.be/cev/avd/files/software/rega_algorithm/Rega_HIV2_Rules_v8.0.2.pdf) [23–25]. The third, Stanford University’s HIVdb Program for HIV-2 (beta) (available at https://hivdb.stanford.edu/hivdb/hiv2/by-sequences/), is based on a statistical analysis of sequences from ART-treated vs. untreated PLWH2 to identify “treatment-selected” mutations [26].

To elucidate the phenotypic effects of mutations in HIV-2 protease that are associated with PI-based ART and evaluate the existing genotypic drug resistance tools, we constructed a library of 54 full-length site-directed protease mutants of HIV-2_ROD9_. This library included all single amino acid substitutions and most of the paired amino acid substitutions that have been identified as treatment-selected or drug-resistance mutations in the Stanford, HIV2EU, and Rega HIV-2 drug resistance algorithms, as well as several mutations identified in cohort studies [27,28]. In addition, we constructed eight multiply-substituted strains containing combinations of mutations observed in PI-treated PLWH2. We determined the susceptibility of each mutant HIV-2_ROD9_ strain to the two HIV-2-active, commercially-available PI, darunavir (DRV) and lopinavir (LPV).

## Methods

### Ethics statement

Protease sequences from PLWH2 were obtained from participants enrolled in prospective cohort studies of ART for HIV-2-infected patients in Senegal, West Africa, with recruitment and follow-up from November 2005 through May 2025. This study was conducted according to procedures approved by the Institutional Review Boards of the University of Washington (approval STUDY00000228) and the Senegalese National Ethics Committee for Health Research (CNERS, approval # SEN 17/60). All participants provided written informed consent.

### Cell lines, antiretrovirals, and wild-type molecular clones

Immortalized cell lines 293T/17 and MAGIC-5A were purchased from the American Type Culture Collection (Manassas, Virginia) or kindly provided by Dr. Michael Emerman (Fred Hutch Cancer Center; Seattle, Washington), respectively. Cells were maintained at 37°C with 5% CO_2_ in Dulbecco’s modification of Eagle Medium (Mediatech; Manassas, Virginia) supplemented with 10% heat-inactivated fetal bovine serum (Hyclone; Logan, Utah or Sigma-Aldrich; St. Louis, Missouri), 4 mM L-glutamine, 50 U/mL penicillin, and 50 μg/mL streptomycin (Gibco, Life Technologies; Grand Island, New York).

The protease inhibitors DRV and LPV were obtained from the National Institutes of Health HIV Reagent Program. Master stocks (20 mM) were prepared in sterile DMSO and stored at -80°C. DRV was water insoluble; serial dilutions of the drug were prepared as working stocks in sterile distilled water and DMSO to a final DMSO concentration of 10%. Serial dilutions of LPV were prepared as working stocks in sterile distilled water. Working stocks were stored at -20°C. The full-length infectious clone of HIV-2 pROD9 (group A) was obtained from Dr. Michael Emerman.

### Generation of site-directed and recombinant plasmid clones

**Fig 1:**
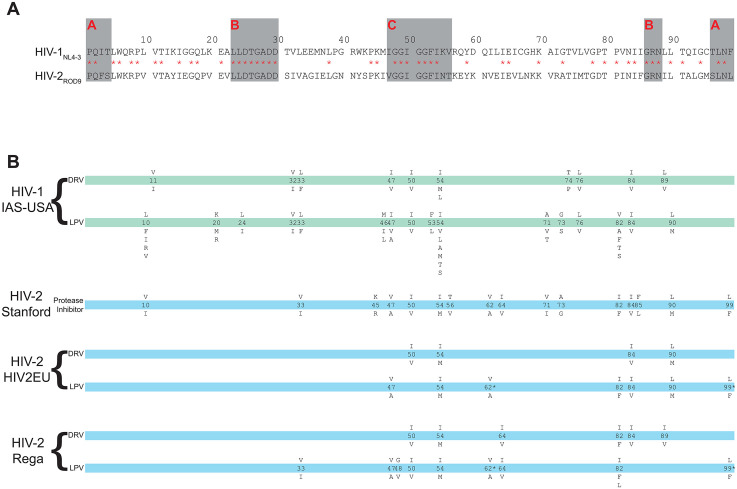
Structural features and putative drug resistance mutations in HIV protease. **(A)** Amino acid alignment of HIV-1_NL4-3_ and HIV-2_ROD9_ protease (adapted from [29]). Identical residues are marked with red asterisks. Grey boxes indicate the boundaries of the dimerization domain **(A)**, active site/carboxy-terminal triad **(B)** and the flap region **(C)** of protease. **(B)** Comparison of putative drug resistance/drug-selected mutations in HIV-2 protease according to three HIV-2 genotypic resistance tools, compared to the IAS-USA mutations in HIV-1 protease. The amino acid occurring in wild-type virus is shown above the codon number, amino acid(s) implicated in drug resistance are shown below. * denotes that both Rega and HIV2EU require V62A and L99F to be observed together to be implicated in lopinavir resistance. Formatting adopted from IAS-USA [30].

We identified single and double amino acid treatment-selected or drug-resistance mutations from the three HIV-2-specific genotypic resistance algorithms: Rega (https://rega.kuleuven.be/cev/avd/files/software/rega_algorithm/Rega_HIV2_Rules_v8.0.2.pdf), HIV2EU (https://www.hiv-grade.de/HIV2EU/deployed/grade.pl?program=hivalg) [23–25], and Stanford HIVdb Program for HIV-2 (https://hivdb.stanford.edu/hivdb/hiv2/by-sequences/) [26] (Fig 1), as well as reports in the published literature [27,28].

HIV-2 clones encoding single amino acid substitutions T56V, V62A, and I64V, as well as clones encoding the combinations I50V+I64V, I54M+T56V, V62A+L99F, and A73G+F85L, were constructed using a megaprimer mutagenesis approach. Briefly, synthetic double-stranded “gBlock” gene fragments (Integrated DNA Technologies; Coralville, Iowa) were designed to include the entire HIV-2 pROD9 protease-encoding sequence, plus 100 base pairs of flanking sequence from each end, with nucleotide changes engineered to introduce the single or multiple amino acid changes of interest. The gBlock fragments were PCR-amplified and purified using the QIAquick PCR Purification Kit (Qiagen; Germantown, Maryland) to increase the amount of starting material. Then, 120 ng of the purified gBlock amplicons were used as “megaprimers” in a second PCR step using 150 ng of pROD9 as the target. PCR steps were performed using Phusion Hot Start Flex DNA Polymerase (New England Biolabs; Ipswich, Massachusetts) according to the manufacturer’s instructions. After the second PCR step, parental plasmid DNA was digested with DpnI (New England Biolabs) and the reaction products were transformed into TOP10 chemically competent *E. coli* (Invitrogen; Waltham, Massachusetts). All other clones containing single and multiple amino acid changes were constructed in full-length pROD9 using the QuikChange II XL Site-directed Mutagenesis Kit (Stratagene; La Jolla, California) per the manufacturer’s instructions, as previously described [31]. All clones were sequenced across the entire genome to ensure no additional mutations were present. All full-length plasmids were purified using a Hi-Speed Plasmid Maxi Kit with endotoxin-free buffers (Qiagen). Nucleotide sequences of the mutagenic primers and gBlocks are available upon request.

### Generation of plasmid clones containing complex combinations of mutations observed in PI-treated Senegalese PLWH2

To test more complex combinations of amino acid changes found in patient-derived viruses, we utilized a recombinant virus approach. Protease sequences from PLWH-2 were obtained from participants enrolled in prospective cohort studies of ART for HIV-2-infected patients in Senegal, West Africa, as described previously [11,31–34], with recruitment and follow-up from November 2005 through May 2025. Briefly, viral nucleic acid was isolated from plasma, PBMCs, or dried blood spots (DBS) by Qiagen kit (QIAamp Viral RNA Mini or DNA Blood, as appropriate for each specimen type), and reverse transcribed using Superscript III (Invitrogen). HIV-2 protease and reverse transcriptase were amplified by nested PCR using MyFi DNA Polymerase mix (Meridian BioScience Inc.; Cincinnati, OH) and sequenced by Sanger dideoxy chain termination methods as described previously [11,31–34]. The resulting nucleotide sequence was translated into amino acids and putative treatment-selected mutations (TSMs) were identified using Stanford’s HIVdb Program for HIV-2. Genotypes of interest, with corresponding immuno-virologic and treatment data, were identified on 6 May 2020. Owing to the relatively low viral loads of HIV-2-infected patients, these “consensus” sequences are likely based on a small number of templates and may contain randomly-occurring nucleotide mutations which are detrimental to viral replication, and which make generating viable clones difficult. To maximize our chances of obtaining replication-competent clones, we opted not to use the exact PLWH2-derived sequence but instead to engineer the combinations of TSMs from each participant into the ROD9 protease. gBlocks were designed encoding the ROD9 protease containing the collection of TSMs from each sequence, and clones were constructed by megaprimer mutagenesis as described above.

### Single-cycle protease inhibitor susceptibility assays

**Fig 2:**
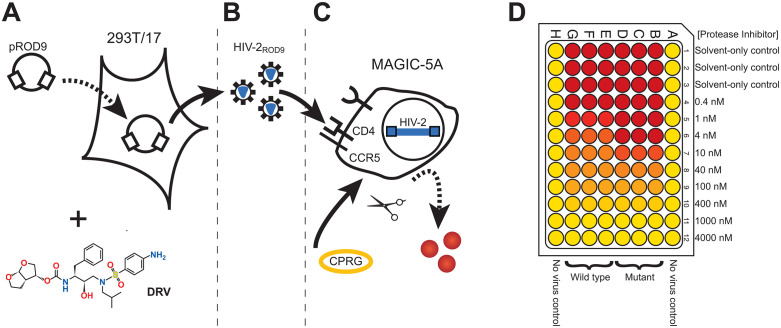
Schematic of PI susceptibility assay. A: Wild type or mutant plasmids are transfected into 293T/17 cells and dosed with protease inhibitors in half-log increments of concentration. B: 293T/17 cell supernatants containing virus are harvested and diluted in DEAE-dextran-containing culture medium. C: Diluted supernatants are plated onto MAGIC-5A indicator cells and viral infection is assayed by CPRG substrate cleavage 46 hours later. D: Example MAGIC-5A plate layout showing controls.

The susceptibilities of each wild-type or mutant HIV-2 molecular clone to LPV and DRV were determined by single-cycle assay, as described previously [31,35] (Fig 2). Briefly, virus stocks were generated by transfecting full-length HIV plasmid DNA into 293T/17 cells using a chloroquine-mediated calcium phosphate method. Twelve hours post-transfection, the media was replaced by fresh medium containing either LPV or DRV in half-log_10_ increments. Due to solubility limits, concentrations of PI greater than 4000 nM were not achievable in the assay. Thirty-six hours later, culture supernatants were harvested, diluted as needed (neet, 1:4, 1:16, or 1:64) to avoid excessive syncytia formation and cytopathic effects (CPE) in DEAE-dextran-containing media, and plated onto MAGIC-5A indicator cells. Approximately 46 hours later, plates were subjected to a β-galactosidase (β-gal) enzyme test using chlorophenol red-β-D-galactopyranoside (CPRG; BioShop Canada; Burlington, Ontario) to assay virus growth, with substrate conversion quantified by measuring absorbance at 570 nm. All CPRG assay values were background-subtracted using A_570_ readings from uninfected culture wells to normalize for intrinsic β-gal activity. For each strain, “% of no-drug control” was calculated by dividing absorbance in drug-treated wells by absorbance in solvent-only treated control wells, then multiplying by 100. Half-maximum effective concentration (EC_50_) values for each strain were calculated from dose-response plots using sigmoidal regression, using a fixed slope model in Prism (version 10.0; GraphPad Software Inc.; San Diego, California). Wild-type HIV-2_ROD9_ was included in each assay run, and all strains were subject to at least three independent EC_50_ determinations (raw EC_50_ values provided in S1 Data). Although formal quantifications of viral titers were not performed, we have previously determined that wild-type HIV-2_ROD9_ produces titers of approximately 100,000 focus-forming units (FFU) per milliliter [31]. Mutant viruses producing titers as low as 100–150 FFU/mL (~0.1% of wild type) can be used for drug sensitivity assays at lower dilutions.

### Statistical analyses

Because of the large number of EC_50_ determinations performed for wild-type HIV-2_ROD9_ versus the relatively small number of tests for each individual mutant strain, we calculated the fold change in EC_50_ by dividing the mean values for each mutant by the mean of the EC_50_ values that were obtained for wild-type HIV-2_ROD9_ in parallel assays (i.e., assays performed on the same date). Pairwise comparisons between mutant and wild-type viruses were performed by first log_10_- transforming the individual EC_50_s, then subjecting the resultant values to two-way independent sample t tests with Welch’s correction for unequal variances. As in the fold change calculations, t tests were done only with wild-type and mutant values that were obtained in parallel. Since all mutations were previously identified as being treatment-selected (and thus, the *a priori* expectation was that many would be resistance-associated), we did not correct for multiple comparisons. Resistance was defined as a mean EC_50_ significantly greater than wild-type, and hypersusceptibility was defined as a mean EC_50_ significantly less than wild-type. We re-analyzed the EC_50_ values for LPV and DRV from our earlier study of HIV protease mutants [31] to ensure that any comparisons between newly-obtained and previously-published data were consistent. Given that the panel of mutant viruses was too large to be able to consistently test strains containing combinations of mutations in parallel with the individual singles, we could not make comparisons between these strains. Statistical analyses were carried out using StataSE (version 14.2; StataCorp.; College Station, Texas).

## Results

We have previously characterized the PI susceptibility of eight HIV-2_ROD9_ strains containing single amino acid changes in protease (V10I, V47A, I54M, V71I, I82F, I84V, L90M, and L99F), as well as six multiply-substituted strains (I54M+I82F, I54M+I84V, I54M+L90M, I82F+L90M, I84V+L90M, and I54M+I84V + L90M) [31]. Since that study was published, two new genotypic resistance algorithms have been developed, which have identified new treatment-associated mutations in HIV-2 protease. In order to fully evaluate the current HIV-2 genotypic drug resistance tools for protease, we tested the remaining thirteen single amino acid changes that are classified as treatment-associated, treatment-selected, or drug resistance mutations in the Rega, HIV2EU, and Stanford drug resistance databases: V33I, K45R, G48V, I50L, I50V, I54L, T56V, V62A, I64V, A73G, A73T, F85L, and I89V, as well as various combinations of mutations which have been identified in those tools. We re-analyzed our previous data using more appropriate statistical methods, allowing for comparisons across data from both studies, including newly-identified multiply-substituted mutant viruses.

Among the 21 single amino acid substituted strains, only four were resistant to DRV (Table 1, bold type) and/or LPV (Table 2, bold type). Compared to wild-type HIV-2_ROD9_, the I50V substitution conferred 4.6-fold resistance to DRV, while the V47A and T56V substitutions conferred 4.1-fold and 2.9-fold resistance to LPV, respectively. Substitution I54M conferred 5.5-fold resistance to DRV and 2.2-fold resistance to LPV, making it the only single amino acid substitution tested to confer resistance to both PI. These substitutions will henceforth be referred to as drug resistance mutations (DRM).

**Table 1:** Susceptibility of HIV-2_ROD9_ treatment-selected mutants to darunavir.

Mutant	Mean EC_50_ ± SD (nM)	n	Fold Change*	t test *p* vs. ROD9
ROD9 wild type	130 ± 88	51		
Single mutations				
V10I^&^	40 ± 17	4	0.91	0.68
V33I	110 ± 52	5	0.85	0.40
*K45R*	*53 ± 15*	*5*	*0.42*	*0.002*
V47A^&^	110 ± 89	6	1.5	0.46
*G48V*	*5.1 ± 1.6*	*4*	*0.082*	*0.0001*
*I50L*	*12 ± 7*	*4*	*0.091*	*0.0001*
**I50V**	**410 ± 310**	**6**	**4.6**	**0.02**
I54L	UTD			
**I54M**^&^	**410 ± 390**	**6**	**5.5**	**0.01**
T56V	240 ± 170	4	1.8	0.54
V62A	230 ± 180	4	1.7	0.40
I64V	160 ± 110	6	1.2	0.75
V71I^&^	64 ± 42	5	0.79	0.42
A73G	180 ± 89	3	1.2	0.66
A73T	110 ± 28	4	0.79	0.31
*I82F*^&^	*12 ± 0.79*	*4*	*0.27*	*0.004*
I84V^&^	54 ± 15	3	1.4	0.21
F85L	140 ± 11	3	1.0	0.77
I89V	120 ± 60	4	0.93	0.69
L90M^&^	160 ± 150	5	2.0	0.35
L99F^&^	46 ± 23	3	1.2	0.71
Non-DRM combinations				
V33I+I64V	93 ± 52	5	0.74	0.20
K45R+L99F	110 ± 60	5	0.90	0.53
V62A+L99F	230 ± 31	3	0.88	0.82
I64V+I84V	670 ± 550	3	4.2	0.21
*A73G+I82F*	*21 ± 9.1*	*2*	*0.069*	*0.004*
A73G+F85L	240 ± 44	5	0.97	0.95
I82F+L90M^&^	41 ± 21	3	0.54	0.28
**I84V+L90M**^&^	**260 ± 96**	**4**	**3.0**	**0.008**
*V10I+I82F + L99F*	*10 ± 9.4*	*3*	*0.17*	*0.03*
V47A combinations				
V47A+V33I	350 ± 230	4	2.2	0.12
V47A+K45R	80 ± 36	4	0.54	0.11
V47A+L90M	160 ± 79	5	0.99	0.79
V47A+L99F	85 ± 88	4	0.97	0.76
**I50V combinations**				
I50V+V33I	1300 ± 1100	5	7.2	0.06
I50V+I64V	300 ± 270	4	2.0	0.41
I50V+I82F	UTD			
**I50V+I84V**	**1100 ± 1500**	**9**	**7.6**	**0.02**
I50V+L90M	700 ± 220	3	3.3	0.05
**I54M combinations**				
**I54M+A73G**	**1900 ± 1500**	**4**	**7.1**	**0.01**
I54M+I82F^&^	87 ± 74	4	0.98	0.63
**I54M+I84V**^&^	**520 ± 170**	**4**	**5.5**	**0.0009**
**I54M+L90M**^&^	**360 ± 210**	**4**	**4.2**	**0.01**
I54M+V10I + V71I	110 ± 36	3	1.9	0.12
I54M+V71I + I82F	140 ± 130	8	0.78	0.35
**I54M+I84V + L90M**^&^	**760 ± 340**	**3**	**14**	**0.02**
T56V combinations				
T56V+I64V	170 ± 67	3	0.8	0.62
T56V+I82F	180 ± 120	5	0.68	0.24
**T56V+I84V**	**2300 ± 1200**	**4**	**12**	**0.0009**
Multi-DRM combinations				
**I50V+I54M**	**4200 ± 2200**	**3**	**16**	**0.002**
**I54M+T56V**	**2100 ± 450**	**3**	**7.8**	**0.003**
V47A+I50V + L90M	UTD			
I50V+I54M + L90M	UTD			
V47A+I50V + I54M+L90M	UTD			

**Bold** **type**: resistant. *Italic type*: hypersusceptible. UTD: unable to determine. * Fold change calculated compared to wild-type EC_50_ conducted in parallel assays. ^&^ Previously published data [31].

**Table 2:** Susceptibility of HIV-2_ROD9_ treatment-selected mutants to lopinavir.

Mutant	Mean EC_50_ ± SD (nM)	n	Fold Change*	t test *p* vs. ROD9
ROD9 WT	130 ± 68	49		
Single mutations				
V10I^&^	100 ± 74	7	0.89	0.32
V33I	130 ± 97	5	1.5	0.61
K45R	44 ± 25	3	0.43	0.09
**V47A** ^&^	**650 ± 150**	**8**	**4.1**	**<0.0001**
*G48V*	*16 ± 6.8*	*5*	*0.13*	*0.0001*
*I50L*	*19 ± 8.7*	*3*	*0.19*	*0.005*
I50V	160 ± 65	7	1.1	0.72
I54L	UTD			
**I54M** ^&^	**300 ± 92**	**8**	**2.2**	**0.0003**
**T56V**	**260 ± 190**	**5**	**2.9**	**0.04**
V62A	160 ± 130	5	1.6	0.57
*I64V*	*18 ± 7.3*	*3*	*0.30*	*0.01*
V71I^&^	69 ± 35	3	0.63	0.52
A73G	120 ± 85	4	1.1	0.95
A73T	92 ± 83	4	0.86	0.55
I82F^&^	160 ± 110	7	1.4	0.62
I84V^&^	140 ± 39	7	1.2	0.18
F85L	220 ± 67	3	1.7	0.12
I89V	120 ± 85	5	1.1	0.88
L90M^&^	220 ± 120	8	1.6	0.10
L99F^&^	150 ± 110	7	1.3	0.83
Non-DRM combinations				
V33I+I64V	220 ± 60	4	1.5	0.10
*K45R+L99F*	*60 ± 27*	*5*	*0.43*	*0.004*
V62A+L99F	200 ± 120	3	2.3	0.15
**I64V+I84V**	**310 ± 93**	**3**	**4.1**	**0.0007**
A73G+I82F	120 ± 130	4	0.95	0.81
A73G+F85L	150 ± 62	4	2.0	0.10
I82F+L90M^&^	180 ± 74	3	1.6	0.31
I84V+L90M^&^	360 ± 140	4	2.4	0.06
V10I+I82F + L99F	130 ± 84	4	0.87	0.65
**V47A combinations**				
**V47A+V33I**	**850 ± 150**	**4**	**5.7**	**<0.0001**
V47A+K45R	280 ± 240	5	2.6	0.25
**V47A+L90M**	**670 ± 600**	**5**	**5.3**	**0.009**
**V47A+L99F**	**530 ± 280**	**5**	**4.4**	**0.002**
I50V combinations				
I50V+V33I	160 ± 74	4	1.2	0.49
I50V+I64V	100 ± 48	4	1.1	0.91
I50V+I82F	UTD			
I50V+I84V	150 ± 43	7	1.2	0.30
I50V+L90M	130 ± 37	3	1.3	0.32
**I54M combinations**				
**I54M+A73G**	**410 ± 200**	**4**	**4.8**	**0.003**
I54M+I82F^&^	330 ± 280	4	2.9	0.18
I54M+I84V^&^	220 ± 140	4	1.6	0.31
**I54M+L90M** ^&^	**610 ± 200**	**5**	**4.5**	**0.002**
I54M+V10I + V71I	110 ± 67	4	0.77	0.50
**I54M+V71I + I82F**	**460 ± 210**	**4**	**3.1**	**0.017**
**I54M+I84V + L90M** ^&^	**530 ± 83**	**3**	**3.6**	**0.0008**
**T56V combinations**				
T56V+I64V	180 ± 61	3	1.8	0.12
**T56V+I82F**	**1200 ± 770**	**5**	**17**	**<0.0001**
**T56V+I84V**	**470 ± 75**	**3**	**5.7**	**0.0003**
Multi-DRM combinations				
I50V+I54M	260 ± 150	3	1.8	0.39
**I54M+T56V**	**440 ± 260**	**3**	**4.0**	**0.03**
V47A+I50V + L90M	UTD			
I50V+I54M + L90M	UTD			
V47A+I50V + I54M+L90M	UTD			

**Bold** **type**: resistant. *Italic type*: hypersusceptible. UTD: unable to determine. * Fold change calculated compared to wild-type EC_50_ conducted in parallel assays. ^&^ Previously published data [31].

A number of single substitutions identified by the three resistance tools or reported in the literature conferred hypersusceptibility to one or both PI, as defined by a mean EC_50_ less than wild-type (Tables 1 and 2, italicized type). Substitutions K45R, G48V, I50L, and I82F conferred hypersusceptibility to DRV, with EC_50_s that were 42%, 8.2%, 9.1%, and 27% of wild-type, respectively. Similarly, substitutions G48V, I50L, and I64V conferred hypersusceptibility to LPV, with EC_50_s that were 13%, 19%, and 30% of wild-type, respectively. Eleven single substitutions had no detectable effect on DRV or LPV susceptibility, and one strain, encoding the I54L substitution, had titers that were too low (estimated at <0.01% of wild type) to determine PI susceptibility.

All three resistance tools identified several combinations of the 21 single amino acid substitutions in protease which occurred together at significantly higher rates among PI-treated PLWH2 compared to untreated PLWH2 [23–26]. We therefore constructed HIV2_ROD9_ variants containing these combinations and tested them for resistance to LPV and DRV in our single-cycle assay (Tables 1 and 2).

No combinations of substitutions that included V47A were found to be resistant to DRV, however the combinations of V47A+V33I, V47A+L90M, and V47A+L99F all conferred significant resistance to LPV, with EC_50_s that were 4.4- to 5.7-fold higher than wild-type. In contrast, the combination V47A+K45R conferred no resistance to LPV. Similarly, strains containing substitutions I82F or I84V in combination with T56V were resistant to LPV (17-fold and 5.7-fold, respectively), while T56V+I64V was not. The combination of T56V+I84V conferred 12-fold resistance to DRV, although neither T56V nor I84V alone conferred DRV resistance. Among strains containing I50V plus another substitution, only I50V+I84V conferred resistance to DRV (7.6-fold).

Substitution I54M, which conferred resistance to both DRV and LPV by itself, is reported in several combinations of treatment-selected pairs or combinations of substitutions. The combination of I54M+A73G conferred significant resistance to both PI (7.1-fold vs. DRV, 4.8-fold vs. LPV), while the combination of I54M+I84V only conferred resistance to DRV (5.5-fold, relative to wild-type), at a level similar to what was observed for I54M alone (Tables 1 and 2) The combination of I50V+I54M conferred 16-fold resistance to DRV, while the combination of I54M+T56V conferred 7.8-fold resistance to DRV as well as 4.0-fold resistance to LPV. In addition, the combination of I54M+V71I + I82F conferred 3.1-fold resistance to LPV. Our previously-published combination of I54M+I84V + L90M conferred resistance to both DRV and LPV (14-fold and 3.6-fold, respectively) [31].

To further investigate the contributions of substitutions which did not confer resistance, we generated mutant HIV-2_ROD9_ strains containing two to four substitutions. Strains containing A73G+I82F and V10I+I82F + L99F conferred hypersusceptibility to DRV, with EC_50_s that were 6.9% and 17% of wild-type, respectively, and the strain containing K45R+L99F conferred hypersusceptibility to LPV, with an EC_50_ that was 43% of wild-type. By comparison, I84V+L90M conferred 3.0-fold resistance to DRV, and I64V+I84V conferred 4.1-fold resistance to LPV.

While strains containing combinations of one DRM and one or two other changes replicated well enough allow determination of drug susceptibility, most combinations containing two or more DRM did not, with clones V47A+I50V + L90M, I50V+I54M + L90M, and V47A+I50V + I54M+L90M resulting in viruses which produced titers too low to assay (<0.01% of wild-type). Since combinations of these changes are observed in highly treatment-experienced PLWH2, this suggests a possible role for TSMs which do not result in resistance. We built HIV-2_ROD9_ virus clones that contained more complicated mutational patterns observed in our cohort of ART-treated PLWH2 in Senegal, West Africa, in an otherwise ROD9 protease backbone (Table 3) [31,34]. These participants had viral loads from 47 to 7510 copies/ml of plasma, and CD4 counts from 50 to 513 cells/μl. All participants were receiving boosted LPV-based ART at the time of sample collection for genotyping, but five of the eight participants had previously received other PI, including indinavir, atazanavir, and/or DRV. Corresponding HIV-2_ROD9_ variants containing up to nine of the 21 individual changes were constructed and studied. Six of our eight clones produced replication-competent viruses, each containing one or two DRM as well as up to six additional substitutions (Table 3). Five of the six strains were highly resistant to LPV (EC_50_s > 8.8 to >20-fold) (Table 4), with the last being 2.1-fold resistant. Three of the six, including two clones that encoded combinations of mutations that were seen in participants who had never received a DRV-containing regimen, were resistant to DRV (EC_50_s 4.7 to 10-fold resistant relative to wild-type). As a result of the solubility limits, exact EC_50_s could not be calculated in all cases; instead, EC_50_s of >1000 nM or >4000 nM were obtained for some mutant strains.

**Table 3:** Clinical data and genotypes of HIV-2 protease sequences from PLWH2 in Senegal.

Study Participant	GenBank Accession Number	Viral Load (copies/ml)	CD4 Count (cells/μl)	Current (previous) PI treatment	Treatment-Selected Mutations
H2A010	MT992803.1	126	392	LPV/r (IDV)	V33I+K45R + **V47A**+V71I + A73G+F85L + I89V+L90M + L99F
H2A059	MT992841.1	1638	418	LPV/r (IDV)	V10I+V33I + **I50V**+**T56V** + I64V+V71I + I82F
H2A064	MT992846.1	2595	165	LPV/r (IDV, LPV/r, DRV)	V10I+V33I + **V47A**+V71I + I82F+I89V
H2A075	PX634191	7510	50	LPV/r (IDV, LPV/r, ATV)	V10I+V33I + **I54M**+**T56V** + V71I+A73G + I82F+L99F
H2A099	PX634192	2919	513	LPV/r	**V47A**+**T56V** + I64V
H2A108	MT992863.1	47	254	LPV/r	V10I+V33I + K45R+**V47A** + **I50V**+F85L + I89V
H2A109	MT992870.1	TND	66	LPV/r (LPV/r, DRV)	V33I+**V47A** + **T56V**+I64V + L90M
H2A124	MT992874.1	180	265	LPV/r	V10I+V33I + **I50V**+I64V

TND: test not done. LPV/r: ritonavir-boosted lopinavir. DRV: darunavir. IDV: indinavir. ATV: atazanavir. **Bold type**: DRM.

**Table 4:** Darunavir and lopinavir susceptibility of HIV-2_ROD9_ variants corresponding to Senegalese patient sequences.

	Darunavir				Lopinavir			
Study participant	Mean EC_50_ (nM)	n	Fold change	p	Mean EC_50_ (nM)	n	Fold Change	p
H2A010	UTD				UTD			
H2A059	1900 ± 1900	5	>11	0.07	**2400 ± 1500**	**6**	**>14**	**0.0002**
H2A064	160 ± 150	5	0.92	0.55	**3000 ± 1300**	**5**	**>16**	**<0.0001**
H2A075	**950 ± 320**	**5**	**4.7**	**0.0004**	**2700 ± 1400**	**5**	**>14**	**<0.0001**
H2A099	320 ± 130	5	1.6	0.06	**1600 ± 630**	**6**	**>8.8**	**<0.0001**
H2A108	UTD				UTD			
H2A109	**1000 ± 890**	**5**	**5.2**	**0.03**	**4100 ± 880**	**4**	**>20**	**0.0003**
H2A124	**2000 ± 1200**	**5**	**10**	**0.006**	**400 ± 26**	**5**	**2.1**	**0.02**

**Bold type**: statistically resistant. UTD: unable to determine.

## Discussion

In contrast to HIV-1, few data exist to guide second-line therapy decisions for PLWH2, and the existing HIV-2 genotypic resistance algorithms are not supported by robust clinical or phenotypic resistance datasets. Our aim was to explore the phenotypic drug susceptibility impacts of the amino acid substitutions in HIV-2 protease which have been identified by existing tools as drug resistance or treatment-selected, in order to more fully understand their contribution to drug resistance.

Studies of HIV-1 have implicated amino acid changes at a large number of sites (~30, depending on the algorithm) in protease inhibitor resistance, including fifteen classified as “major” resistance-associated sites by the International AIDS Society [30]. The majority of the 18 codon sites identified by the HIV-2 genotypic resistance tools are sites that have also been implicated in PI resistance in HIV-1 (Fig 1). We have previously published PI resistance data for several mutations implicated by these tools, and chose to re-analyze and include the old data to allow fair comparisons between the old data and the new. We felt this was particularly important given the number of newly-identified combinations of mutations which include single mutants that we had studied previously.

In our single-cycle phenotypic drug resistance assay, approximately half of the single amino acid substitutions identified by one or more HIV-2 genotypic tools conferred no change in EC_50_ to either LPV or DRV by themselves, and five additional substitutions conferred hypersusceptibility to one or both drugs (Tables 1 and 2). These findings suggest that although the mutations listed in the genotypic resistance tools or algorithms for HIV-2 protease are treatment-selected, they may not directly contribute to PI resistance. Instead, they likely play some other role, possibly compensating for a loss of viral fitness incurred by other resistance-conferring changes, or enhancing the drug resistance effects of other mutations. Mutations in HIV-1 protease at codons 10, 33, 73, and 89 have been shown to reduce sensitivity to various PI either alone or in combination, but we found no evidence suggesting that the same is true in HIV-2, at least for DRV or LPV. Mutations K45R, I64V, F85L, and L99F have no analogous HIV-1 changes that have been implicated in drug resistance, suggesting that these may be novel treatment-selected changes. However, this latter group appears more likely to be compensatory or accessory mutations rather than contributing directly to drug resistance (Tables 1 and 2). Tzou *et al.* noted that changes V10I, I64V, V71I, and L99F were observed in PI-naïve patients, although they were found at higher rates among PI-treated PLWH2, and thus described them as polymorphic treatment-selected changes [26].

Substitution G48V, which in HIV-1 causes resistance to saquinavir, and substitutions I50L and I54L, which in HIV-1 cause resistance to atazanavir, have been reported infrequently [28,36], with I50L and I54L reported in PLWH2 treated with atazanavir. Saquinavir is no longer commercially available, and atazanavir is not clinically useful against HIV-2 [28]. Mutations G48V and I50L do not seem to cause resistance to DRV or LPV, however their impacts against pipeline PI may be more important. We were unable to test I54L so its contribution to PI resistance remains unknown. Mutations V62A and L99F, reported among IDV-treated PLWH2 either alone or in combination [27,31,32,37], do not appear to cause resistance to either DRV or LPV alone, but together conferred a non-significant increase in EC_50_ to LPV, and warrant further study. Although none of these changes cause resistance to the two PI tested herein, as novel PI are developed, it will be important to test these mutations for resistance against the new drugs.

Codon 84 is a site of “major” resistance changes in HIV-1, but mutation I84V did not confer significant resistance in HIV-2 by itself. It is possible that I84V confers an effect too small to detect in our assay. Some I84V combinations, where neither mutation caused significant resistance alone, e.g., I84V+L90M or I64V+I84V, resulted in increases in EC_50_ to DRV and LPV (2.4 to 4.2-fold). Some, but not all, of these increases were statistically significant in our assay, hinting at a possible direct resistance role for these changes. The mechanism of this potential action is unknown but may include, for example, subtle changes to the enzyme active site which are insufficient alone to prevent PI binding, but in combination cause a more pronounced effect.

We have previously shown [31], and demonstrate here again using different statistical methods, that the isoleucine to phenylalanine substitution at codon 82, also a site of “major” resistance changes in HIV-1, not only confers no resistance, but in fact appears to confer hypersusceptibility to PI in HIV-2. Although I82F is treatment-selected in PLWH2 [31,32,36,38–44] and emerges during serial passage selection experiments [45], no study to date has provided phenotypic evidence that this change alone, in a fully wild-type HIV-2 backbone, cause drug resistance. However, the substantial increase in EC_50_ against LPV that we observed in this study when I82F is added to T56V, with or without other TSMs, warrants further investigation as a possible unique HIV-2 resistance pathway. We have previously shown that I82F causes a dramatic drop in viral replication capacity [31], making it less likely to be a compensatory fitness mutation. We and others have demonstrated that codon 82 is an important determinant in PI susceptibility differences between HIV-1 and HIV-2 [35,46–51], and posit that the role of codon 82 in defining the conformation of the protease active site may explain the frequent observation of the I82F change in PI-treated PLWH2, despite causing no detectable PI resistance. The frequent inclusion of this change as a “drug-resistance” change in HIV-2 genotypic tools is problematic without further mechanistic studies, since it does not appear to cause drug resistance alone or in the majority of combinations we tested.

Four substitutions, V47A, I50V, I54M, and T56V, all of which occur within the flap region of protease, confer resistance to one or both drugs with EC_50_s 2.2-fold to 5.5-fold higher than wild-type, and should be considered true DRM. While single or double amino acid changes in reverse transcriptase or integrase can confer many times higher-fold resistance than we report here for protease [52–55], these fold change values are consistent with those previously published for corresponding mutants of HIV-1 [56]. In HIV-1, changes at protease codons 47, 50, and 54 are considered “major” mutations, conferring resistance to multiple PI, including both DRV and LPV. However, to our knowledge, no previous study has implicated codon 56 in drug resistance in either HIV type, underscoring the utility of looking beyond the known genotype-to-phenotype relationships for HIV-1 when considering drug resistance in HIV-2.

The additive effects of HIV-2 DRMs V47A, I50V, I54M, and T56V proved difficult to study, since the majority of HIV-2_ROD9_ clones carrying two or more of these changes did not produce titers that were sufficient for PI susceptibility testing. However, clones containing highly-substituted protease sequences that were based on our genotypic analysis of PI-treated PLWH2 (Tables 3 and 4) exhibited high-level resistance to DRV and LPV, providing evidence of an additive effect of multiple DRM and further supporting a compensatory fitness role (with or without resistance enhancement) for other treatment-selected changes. Importantly, four of the six variants tested were based on sequences from PLWH2 who had never been treated with DRV, yet two of these were highly resistant to DRV, suggesting significant cross-resistance and the possibility of rapid treatment failure on DRV salvage therapy. Even the strain from patient H2A064, which harbored no mutations known to confer DRV resistance, was 4.7-fold resistant to DRV relative to wild-type, suggesting that cross-resistance may complicate salvage therapy.

We used titer estimations as well as direct observations of CPE to guide virus dilutions. All plates were visually inspected for CPE and plates with excessive syncytia formation which would have artificially inflated EC_50_ values were excluded. We have previously found that, for NRTI, multiplicity of infection (MOI) has a minimal impact on inhibition levels in a similar assay [54], and that syncytia formation and cytopathic effects have a greater impact on PI EC_50_. Although analyses of replication capacity were outside of the scope of this study, such data could yield additional insights into the fitness of particular mutant viruses, further elucidating the roles of mutations which were not observed to cause PI resistance.

Our study has several limitations. First, our single-cycle PI susceptibility assay has two cell culture steps involving different parts of the viral life cycle, which occur in different cell lines: a virus production step in PI-treated 293T/17 cells, and an infection and scoring step in MAGIC-5A cells. As a result, there is more assay “noise” than an assay in which there is only one culture step. The impact of this can be observed in the high standard deviations we report, and some low-level resistance signals may be lost as a result. Second, it would be difficult, if not impossible, to make and test all possible combinations of the 21 single amino acid changes studied. However, understanding the interactions between each of the changes could provide clues to which “accessory” mutations are purely compensatory, versus which confer low-level resistance alone or enhance the level of resistance conferred by other mutations. It would also be very helpful to be able to compare EC_50_s from combinations of mutations to their parent single mutations to look for additive or multiplicative effects, but there are simply too many combinations to make that strategy feasible given current technology and fiscal constraints. Additionally, the impact of mutations in the HIV-2 *gag*, which may play a role in resistance to PI, have been the topic of only one study of which we are aware [57], and may represent important mechanisms of resistance. Finally, and critically, all of our existing SDM phenotypic drug susceptibility work has been conducted in HIV-2_ROD9_, which is a group A strain. Only one phenotypic HIV-2 drug resistance study to date has examined the impacts of any mutations – most of which have not been subsequently implicated in PI resistance – in an HIV-2 group B virus backbone. The assumption of similarity between analogous mutations across the two groups is entirely untested.

Decades’ worth of clinical experience treating PLWH, as well as laboratory studies of genotypic and phenotypic HIV drug resistance, have proven unequivocally that HIV-1 and HIV-2 do not necessarily respond the same to antiretroviral therapy [58–61]. HIV-2 protease is only 50% identical to HIV-1 protease at the amino acid level, and is intrinsically resistant to more than half of US Food and Drug Administration- (FDA-) approved PI, so it should come as no surprise that genetic pathways to PI resistance can differ between HIV-1 and HIV-2. Our results highlight the dangers of extrapolating from HIV-1 data without performing confirmatory phenotypic testing in HIV-2, since there are a number of substitutions, including a possible significant drug resistance mutation, with no analogous HIV-1 changes. Given the possibility of TLD failure in PLWH2 and the potential need for PI-based second-line or salvage therapy, more work is urgently needed in this area. Our data add to our understanding of protease inhibitor resistance in HIV-2, allowing for refinement of existing genotypic resistance tools for interpreting sequences from HIV-2-infected patients, and may help to inform treatment decisions for PLWH2 in resource-limited settings.

## Supporting information

S1 Data Susceptibility of HIV-2_ROD9_ treatment-selected mutants to darunavir and lopinavir, raw EC_50_ values (nM). All data with harvest dates prior to 2014 were previously published [31].
(XLSX)
